# Antibacterial Efficacy of Two Commercially Available Bacteriophage Formulations, Staphylococcal Bacteriophage and PYO Bacteriophage, Against Methicillin-Resistant *Staphylococcus aureus*: Prevention and Eradication of Biofilm Formation and Control of a Systemic Infection of *Galleria mellonella* Larvae

**DOI:** 10.3389/fmicb.2020.00110

**Published:** 2020-02-07

**Authors:** Tamta Tkhilaishvili, Lei Wang, Arianna Tavanti, Andrej Trampuz, Mariagrazia Di Luca

**Affiliations:** ^1^Center for Musculoskeletal Surgery, Charité – Universitätsmedizin Berlin, Berlin Institute of Health, Corporate Member of Freie Universität Berlin, Humboldt-Universität zu Berlin, Berlin, Germany; ^2^Berlin-Brandenburg Center for Regenerative Therapies, Charité – Universitätsmedizin Berlin, Berlin, Germany; ^3^Department of Biology, University of Pisa, Pisa, Italy

**Keywords:** methicillin-resistant *Staphylococcus aureus*, biofilm-associated infection, antimicrobial activity, bacteriophages, *Galleria mellonella*, phage therapy, isothermal microcalorimetry, confocal laser scanning microscopy

## Abstract

Sessile bacteria growing on surfaces are more resistant to standard antibiotics than their planktonic counterpart. Due to their antimicrobial properties, bacteriophages have re-emerged as a promising approach to treat bacterial biofilm-associated infections. Here, we evaluated the ability of two commercially available phage formulations, Staphylococcal bacteriophage (containing the monophage Sb-1) and PYO bacteriophage (a polyphage), in preventing and eradicating an *in vitro* biofilm of methicillin-resistant *Staphylococcus aureus* (MRSA) by isothermal microcalorimetry and high-resolution confocal laser scanning microscopy (CLSM). Moreover, to assess the potential *in vivo* efficacy of both phage preparations, a *Galleria mellonella* model of MRSA systemic infection was used. Microcalorimetry measurement showed that 10^7^ PFU/ml (the highest tested titer) of both phage formulations were able to inhibit planktonic growth in a concentration-dependent manner. However, MRSA biofilm was eradicated only by co-incubation of 5–7 days with the highest phage titers, respectively. In the experiments of biofilm prevention, isothermal microcalorimetry revealed that the heat production was completely abolished in the presence of sub-inhibitory titers (10^4^ PFU/ml) of phages. These data were also confirmed by confocal laser scanning microscopy. Both phage formulations increased the survival of *G. mellonella* larvae preventing or treating MRSA infection compared to untreated control. In conclusion, tested phage formulations are promising for preventing device colonization and killing biofilm bacteria attached on a surface. Novel strategies for direct coating and release of phages from material should be investigated.

## Introduction

*Staphylococcus aureus* is causing a variety of community-acquired and healthcare-associated infections (Magill et al., 2014; Tong et al., 2015). In addition, *S. aureus* exhibits the ability to form biofilm on either native tissues or implanted medical devices resulting in tolerance to high concentrations of antimicrobials (Zimmerli et al., 2004; Arciola et al., 2012). Infections caused by biofilm-embedded bacteria are difficult to eradicate due to an extracellular polymeric matrix, which protects them from antimicrobials and host immune cells (de la Fuente-Nunez et al., 2013; Paharik and Horswill, 2016). Indeed, the heterogeneity of the biofilm cell populations, including antibiotic-tolerant persister cells, characterized by a slow- or non-growing state, makes biofilm-embedded bacteria significantly less susceptible to antimicrobials than their free-floating counterparts (Van Acker et al., 2014; Flemming et al., 2016). Moreover, the emergence of spreading of staphylococcal strains resistant to different antimicrobial agents, including methicillin, vancomycin, daptomycin and/or rifampicin (O’Neill et al., 2006; Kos et al., 2012; Hassoun et al., 2017; Ma et al., 2018) represents a serious threat to global health (Sugden et al., 2016; Monaco et al., 2017).

This scenario is further complicated by the fact that production pipelines for the development of novel antibiotics has been running dry over the past few decades, resulting in an crucial requirement to identify novel therapeutic strategies to control bacterial infections mainly due to multi-drug resistant bacteria embedded in a biofilm (Ribeiro et al., 2016).

Bacteriophage (phage) therapy, based on the employment of viruses specifically killing bacterial cells, is considered an encouraging option for treating staphylococcal infections which result refractory to conventional antibiotics (Gordillo Altamirano and Barr, 2019). After the discovery of bacteriophages by d’Herelle in 1917, phages as therapeutic agents have been used for almost century mainly in the Eastern European countries in humans (Kutateladze and Adamia, 2010). However, with the launching of conventional antibiotics, the application of phage therapy in Western countries promptly decreased (Kortright et al., 2019). The rapid increase of multi-drug-resistant bacterial strains recently has been renewed interest in phage therapy and even though regulatory authorities in Europe and U.S. have not approved it yet, several examples of successfully applications of personalized phage therapy have been reported (Jennes et al., 2017; Chan et al., 2018; Exarchos et al., 2019; Nir-Paz et al., 2019; Tkhilaishvili et al., 2019) as compassionate use under the umbrella of Article 37 of the Helsinki Declaration.

Phage therapy offers some advantages over conventional antimicrobial strategies. Due to their high specificity, bacteriophages attack only host bacterial cells without affecting the normal microflora (Ly-Chatain, 2014). Unlike antibiotics, bacteriophages are self-propagating and self-limiting viruses, regulating themselves at the site of infection. This behavior results in a localized increase in viral particle numbers with a low initial dose as long as the targeted bacteria are present and in a decrease when bacteria have been killed (Dabrowska, 2019). Moreover, since the resistance mechanisms arose for all class of antibiotics do not alter phage infection, phages have been demonstrated to kill multidrug-resistant bacterial cells.

In addition, for different phages, the ability to reduce *in vitro* viable sessile bacterial cells was also proved (Khalifa et al., 2015; Liu et al., 2016; Kumaran et al., 2018), suggesting a potential use for the treatment of biofilm-associated infections.

Among commercially available phage formulations for therapeutic use in human, Staphylococcal bacteriophage (Sb) and PYO bacteriophage (PYO) were developed and employed as anti-infective treatments at Eliava Institute in Georgia (Fish et al., 2018; Ujmajuridze et al., 2018). Sb is a mono phage preparation that contains a well characterized and fully sequenced virus Sb-1 (Kvachadze et al., 2011), whereas PYO is a complex preparation targeting different bacterial species including *S. aureus*, *Streptococcus* spp., *E. coli*, *Pseudomonas aeruginosa*, and *Proteus* spp. with batch to batch variations (Kvachadze et al., 2011; Villarroel et al., 2017).

Sb has been successfully employed to treat *S. aureus* infections in different patients suffering from digital osteomyelitis and foot ulcers (Fish et al., 2016). Analogously, PYO have been used to cure staphylococcal wound infections by either washing the wound or applying a dressing impregnated with the phage cocktail (Pokrovskaya et al., 1942; Markoishvili et al., 2002).

Recently, we have showed that Sb is able to degrade the components of extracellular matrix and be effective against persister cells of *S. aureus* (Tkhilaishvili et al., 2018b). In addition, this phage formulation exhibited a rapid synergistic activity in eradicating *S. aureus* biofilm after 24 h-treatment in combination with different classes of antibiotics (Tkhilaishvili et al., 2018b).

Although both phage formulations were widely used in Former Soviet Union (Sulakvelidze et al., 2001; Myelnikov, 2018), their lytic effect against sessile bacteria has not been investigated in a preclinical setting yet.

Here, we evaluated the ability of Sb and PYO to prevent and eradicate an *in vitro* biofilm of methicillin-resistant *S. aureus* (MRSA) by isothermal microcalorimetry (IMC) and high-resolution microscopy. In addition, the *in vivo* efficacy of both phage formulations was also evaluated in a *Galleria mellonella* model of *S. aureus* systemic infection.

## Materials and Methods

### Bacterial Strains and Bacteriophages

MRSA ATCC 43300 was used for all experiments. Bacteria were stored in a cryovial bead preservation system (Roth, Karlsruhe, Germany) at −80°C. Bacterial strains were grown on blood agar plate (VWR Chemicals, Leuven, Belgium) at 37°C for 24 h. Inoculum was prepared according to a McFarland (BioMerieux Marcy l’Etoile, France) turbidity of 0.5 (≈1–5 × 10^8^ CFU/ml of the tested strain). Commercially available formulations of staphylococcal bacteriophage and PYO bacteriophage were obtained as 10 ml liquid ampoules from the Eliava Biopreparations, a company associated with the G. Eliava Institute of Bacteriophages, Microbiology and Virology, Tbilisi, Georgia. PYO formulation Phage stocks were maintained at 4°C. Vancomycin was supplied by Teva Pharma AG (Aesch, Switzerland) as 10 mg of powder in ampoules. The stock solution of 50 mg/ml was prepared in sterile saline.

### Titration of Bacteriophage Suspensions

Phages titers were determined by a quantitative plaque assay as previously described (Tkhilaishvili et al., 2018a). Phage formulations were diluted using 10-fold serial dilutions in phosphate buffer to an estimated concentration yielding plaque numbers that could easily be counted. From appropriate dilutions, 0.5 ml bacteriophage lysate and 0.3 ml host bacteria from overnight culture were added to 3 ml top agar (ca. 50°C) and immediately poured onto Brain-Heart Infusion agar plates. Two plates for each dilution were used. After overnight incubation of plates at 37°C, plaques were counted and the titer was calculated in PFU/ml.

### Microcalorimetry Assay

An isothermal calorimetry instrument (Thermal Activity Monitor, Model 3,102 TAM III, TA Instruments, New Castle, DE, USA) equipped with 48 channels was used to determine the antimicrobial activity of bacteriophages, as previously reported (Tkhilaishvili et al., 2018a,b). Airtight sealed ampoules were sequentially introduced into the microcalorimetry channels and lowered to an equilibrium position for 15 min to reach a temperature of 37°C. The heat generated in real-time by planktonic and biofilm-embedded cells treated with phages and by recovering bacteria after treatment were continuously measured. Heat flow (μW) was measured at 120 s-intervals and recorded for either 24 or 48 h.

### Phage Lytic Activity Against Planktonic Methicillin-Resistant *Staphylococcus aureus* by Isothermal Microcalorimetry and CFU Counting

Free-floating bacteria were added to microcalorimetric ampoules containing 3 ml of BHIB (final inoculum 1–5  ×  10^6^ CFU/ml) and 10-fold serial dilutions of phages (ranging from 10^2^ to 10^7^ PFU/ml). A growth control containing bacteria without phages, as well as a negative control with phages only was also included. Bacterial heat production was monitored for 24 h at 37°C and data were plotted as heat flow (in μW) and total heat (J) versus time. The minimum heat inhibiting concentration of phages (MHICP) was defined as the lowest titer inoculated with bacteria at the experiment starting point that inhibited growth-related heat production during 24 h-incubation in the microcalorimeter by more than 90% in comparison to the untreated control (growth control). After calorimetric analysis, 50 μl of the culture and related 10-fold serial dilutions were plated onto BHI agar for colony counting. MBC was defined as the minimum bactericidal titer of phages which determined a reduction of more than 3 log_10_ CFU/ml comparing to the CFU/ml of inoculum size. Experiments were performed in triplicate.

### Phage Lytic Activity Against Biofilm Methicillin-Resistant *Staphylococcus aureus* by Isothermal Microcalorimetry Testing and Sonication/Colony Counting

#### Real-Time Isothermal Microcalorimetry

MRSA biofilms were formed on porous glass beads having a diameter 4 mm, pore size 60 μm, and surface area approximately 60 cm^2^ (VitraPor; ROBU, Hattert, Germany). Briefly, 10 beads were statically incubated with 2–3 colonies of MRSA into 10 ml BHI broth at 37°C. After 24 h incubation, beads were carefully washed three times using sterile PBS and incubated with 10-fold serial dilution phage titers (ranging from 10^2^ to 10^7^ PFU/ml) into the microcalorimetry glass ampoules filled with a final volume of 3 ml fresh BHI broth. Sterile beads and beads with untreated biofilm were also included as a negative (sterility) and positive (growth) control. IMC analyses were recorded for 48 h at 37°C. The minimal heat inhibitory concentration of phages for biofilm (MHICP_biofilm_) bacteria was defined as “the lowest phage titer inhibiting growth-related heat production related to the viability of biofilm cells during 48 h-incubation in the microcalorimeter more than 90% (corresponding more than 2 log_10_-reduction of CFU) compared to the growth control. Experiments were performed in triplicate.”

#### Evaluation of the Eradication of Biofilm Methicillin-Resistant *Staphylococcus aureus* by Isothermal Microcalorimetry

Twenty-four-hour-old biofilms on the beads prepared as described above were co-incubated to the 10-fold serial dilution bacteriophage titers (ranging from 10^2^ to 10^7^ PFU/ml) in plastic FAC tubes (Corning Science, Reynosa, Mexico) for 24–48–72–120–168 h. Then, beads were carefully rinsed (3×) using sterile PBS to remove planktonic bacteria and phages, incubated in ampoules with fresh medium and inserted into the calorimeter for the eradication analysis. Growth medium with untreated beads was used as the positive (growth) control, and growth medium with sterile beads served as the negative (sterility) control. The minimum biofilm bactericidal concentration of phages (MBBCP) was defined as the lowest antimicrobial concentration that strongly reduced the number of viable bacterial cells within the biofilm, and therefore leading to undetectable heat values for 24–48 h. Experiments were performed in triplicates.

#### Biofilm Prevention Assay by Isothermal Microcalorimetry

An inoculum, prepared according to a McFarland standard turbidity of 0.5 was diluted to a final concentration of 1–5 × 10^6^ CFUs/ml, as reported above. Ten beads were incubated with 1–5  ×  10^6^ CFU/ml of MRSA together with 10-fold serial dilution phage titers (ranging from 10^2^ to 10^7^ PFU/ml) into 10 ml BHI broth for 24 h at 37°C in static condition. After 24 h-incubation, beads were carefully rinsed (3×) with sterile PBS and incubated in sterile glass ampoules with 3 ml BHI broth. Sterile beads and beads with untreated biofilm were also here included as a negative (sterility) and positive (growth) control. The IMC analysis was performed at 37°C for 48 h, defining the minimum biofilm preventing concentration (MBPCP) of phages as the lowest phage titer that prevented the formation of biofilm on the glass beads, thus leading to an undetectable heat flow signal during 48 h-incubation in the calorimetry.

#### Sonication/Colony Counting

After IMC biofilm experiments, to evaluate the reduction/eradication of biofilm cells, the beads showing no heat production together with untreated biofilms (growth controls) were washed (3×) using sterile PBS to remove the rest of phages and planktonic bacteria and transferred to individual Eppendorf tubes with 1 ml saline. Beads were vortexed for 30 s with maximum power, sonicated at 40 kHz for 60 s in a sonication bath (BactoSonic; Bandelin Electronic, Germany), and vortexed for 30 s again to dislodge biofilm bacteria. For conventional culture, sonication fluids were serially diluted in Eppendorf tubes and aliquots of 50 μl were quantified by viable count of CFU/ml. In unpublished control experiments, in which *S. aureus* cultures were sonicated up to 30 min (with the same above mentioned conditions), no statistically significant difference in CFU values was observed in comparison to non-sonicated cultures, indicating that under these conditions sonication does not kill bacteria. The minimum biofilm eradicating concentration (MBECP) of phage titers was defined as MBBCP titers, but resulting in 0 CFU/ml on plates after CFU counting of the sonicated beads, as previously described (Tkhilaishvili et al., 2018b).

### Confocal Laser Scanning Microscopy

The lytic effect of phage on the prevention of biofilm formation and its eradication was evaluated by CLSM. Brieflyan overnight bacterial culture (diluted 1:100) was distributed into an 8-well μ-Slide (Ibidi) to form biofilm. For prevention experiments, bacteria were simultaneously incubated (at 37°C for 24 h) in the presence of different titres of phages. For eradication experiments, bacteria were first let form biofilm into an 8-well μ-Slide (Ibidi) for 24 h at 37°C, and then treated with different phage titers. Bacteria viability and biofilm thickness after phage co-incubation/treatment was determined by CLSM after staining cells with Syto9 (488 nm/500–540 nm) and propidium iodide (PI) (561 nm/600–650 nm) as recommended by the manufacturer (Live/dead BacLight Bacterial Viability Kit Molecular Probes, Life technologies). Samples were analyzed by the microscope TCS SP5 (Leica, Heidelberg, Germany) using a 63× objective and a pinhole aperture of 1.0 Airy. For each image, the mean of fluorescent intensity was calculated as previously described.

### Phage Treatment in a *Galleria mellonella* Model of Methicillin-Resistant *Staphylococcus aureus* Infection

Larvae of *G. mellonella* were obtained from BioSystems Technology Ltd. (Exeter, Devon, UK). Larvae were stored at room temperature and were used within 3 days. Phages were tested for their ability to rescue MRSA-infected larvae from death. Bacteria were prepared for injection as previously described (Gibreel and Upton, 2013). Larvae were inoculated with 10 μl of bacterial suspension (containing ≈ 2.5–5 × 10^6^–2.5–5 × 10^7^ CFU) in the last left proleg. For the treatment, phages (1 × 10^5^ PFU) or vancomycin (10 mg/kg) were delivered behind the last proleg on the opposite side to the bacterial injection site either 1 h post-infection (for treatment experiments) or 1 h pre-infection (for prevention experiments). Ten larvae per treatments were issued in all experiments. Larvae infected and treated with PBS solution served as positive control group. Three negative control groups were also included in the experimental design: one group that underwent no manipulation, one group injected with PBS only, which controlled for the impact of any negative effect from the injection process, and one group injected with phage suspension only, assessing phage toxicity. Larvae were stored in Petri dishes in the dark at 37°C for 168 h. Larvae were inspected every 24 h and were considered dead if they did not move when stimulated.

### Data Analysis

Microcalorimetry data analysis was accomplished using the manufacturer’s software (TAM Assistant; TA Instruments, New Castle, DE). Figures were plotted using GraphPad Prism 6.01 (GraphPad Software, La Jolla, CA, USA), and resulted data were expressed as heat flow (μW) and total heat (J) versus time (h). In the *G. mellonella* model of infection, survival data were plotted using the Kaplan-Meier method.

## Results

### Antibacterial Activity of Sb and PYO *versus* Planktonic Methicillin-Resistant *Staphylococcus aureus*

**Figure 1:**
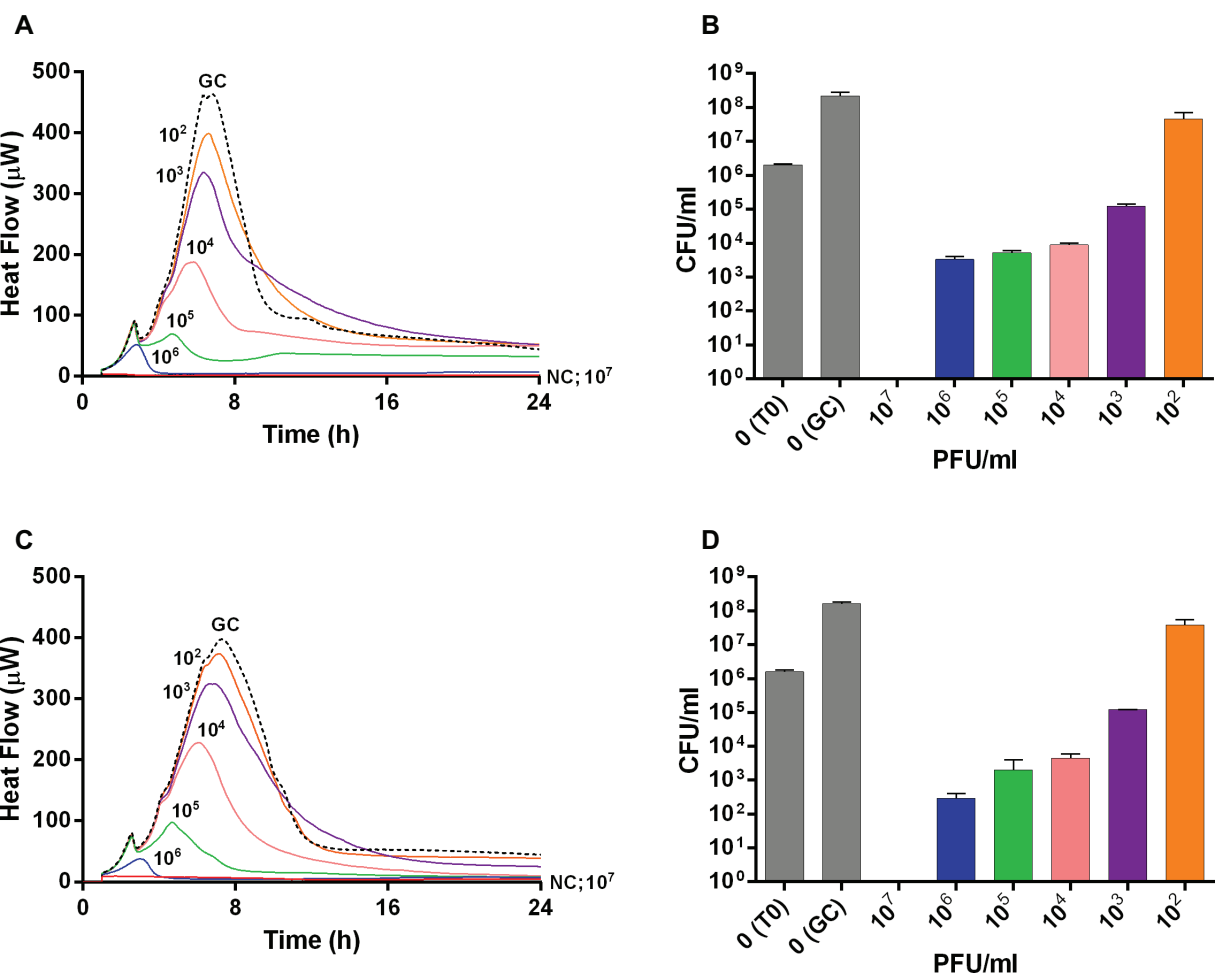
Evaluation of MRSA ATCC43300 susceptibility to Sb **(A,B)** and PYO **(C,D)** exposure, by isothermal microcalorimetry **(A,C)** and colony counting **(B,D)**. Each curve shows the effect of different titers of phages on the heat produced by viable bacteria during 24 h of treatment with Sb **(A)** and PYO **(C)**. Histogram represents the mean of CFU number ± SEM of planktonic MRSA treated/untreated with Sb **(B)** and PYO **(D)**. Numbers above curves represent Sb titers (PFU/ml). GC, growth control (dashed line); NC, negative control; T0, initial inoculum.

The viability of planktonic MRSA was investigated in real-time over 24 h by IMC measuring the heat produced by MRSA in the presence of phages and by CFUs counting after phage treatment (Figure 1). An untreated growth control was also added. As shown in Figures 1A,C, either Sb or PYO rapidly inhibited the planktonic growth of MRSA in a titer-dependent manner compared to the untreated growth control. Indeed, no heat production was observed in the presence of 10^7^ PFU/ml of both phage formulations within 24 h-incubation, indicating that 10^7^ PFU/ml titer corresponds to MHICP.

A similar dose-dependent trend was observed by colony counting of bacteria after 24 h incubation with different phage titers. As shown in Figures 1B,D, an increase of ≈2 log_10_ CFU/ml was observed in the GC samples, as compared to the inoculum size (T0). A reduction of more than 2 log_10_ was already obtained with 10^4^ PFU/ml of Sb and PYO phages, respectively, compared to the CFU/ml number of MRSA initial inoculum (1–5 × 10^6^ CFU/ml). In the presence of 10^7^ PFU/ml titers of both phage formulations, no CFUs were observed (plating detection limit = 20 CFU/ml), suggesting that such titer is MBCP.

### Antibacterial Activity of Sb and PYO Against Biofilm-Embedded Methicillin-Resistant *Staphylococcus aureus*

**Figure 2:**
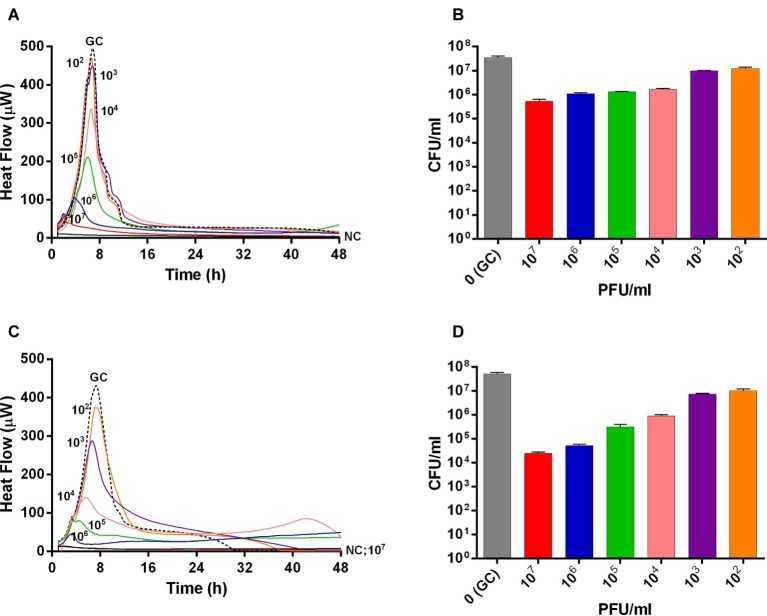
Evaluation of MRSA ATCC43300 biofilm susceptibility to either Sb **(A,B)** or PYO **(C,D)** exposure, by isothermal microcalorimetry **(A,C)** and colony counting **(B,D)**. Each curve shows the heat produced by viable bacteria attached on beads during 48 h treatment with different titers (ranging from 10^2^ to 10^7^ PFU/ml) of Sb **(A)** and PYO **(C)**, respectively. Histogram represents the mean of CFU number ± SEM of biofilm dislodged MRSA treated/untreated with Sb **(B)** and PYO **(D)**. Numbers above curves represent Sb titers (in PFU/ml). GC, growth control (dashed line); NC, negative control.

The interaction between phages and 24 h-old *S. aureus* biofilm was also analyzed in real-time by microcalorimetric measurements. The thermogenic curves of biofilm-embedded cells treated and untreated with phages are shown in Figure 2. Either Sb and PYO inhibited the replication of sessile bacteria in a titer-dependent manner compared to the growth control, resulting in a suppression of the heat production over 48 h-incubation (Figures 2A,C). However, a reduction of more than 90% of the total heat produced by MRSA biofilm-embedded cells was observed at 10^7^ PFU/ml titers for both phage formulations and therefore was defined as MHICP_biofilm_.

**Figure 3:**
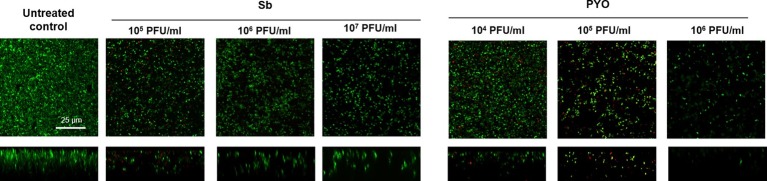
CLSM images of MRSA ATCC 43300 biofilm treated with/without phages. MRSA biofilm (24 h-old) was exposed for further 24 h to different phage titers (10^4^–10^7^ PFU/ml). The viability of the cells was evaluated staining biofilm with SYTO9 (488/500–540 nm – green) for alive bacteria and with propidium iodide (PI) (561/600–650 nm – red) for dead bacteria. Images are merged from the two channels. Upper and lower panels represent xy- and z-plans, respectively. Scale bar: 25 μm.

Then, the evaluation of viable bacteria attached to the beads was performed by colony counting after bead sonication and plating of the sonication fluids. A similar dose-dependent trend of reduction of MRSA CFUs/ml was observed for all samples treated with both phages, as compared to the untreated growth control (Figures 2B,D). Here, the colony counting showed a reduction of more than 2 log_10_ when bacteria were treated with 10^7^ PFU/ml of Sb and more than 3 log_10_ with 10^7^ PFU/ml of PYO phage compared to the growth control (plating detection limit = 20 CFUs/ml). The lack of an eradication at higher titers after 24 h of phage treatment was also confirmed by CLSM (Figure 3).

**Figure 4:**
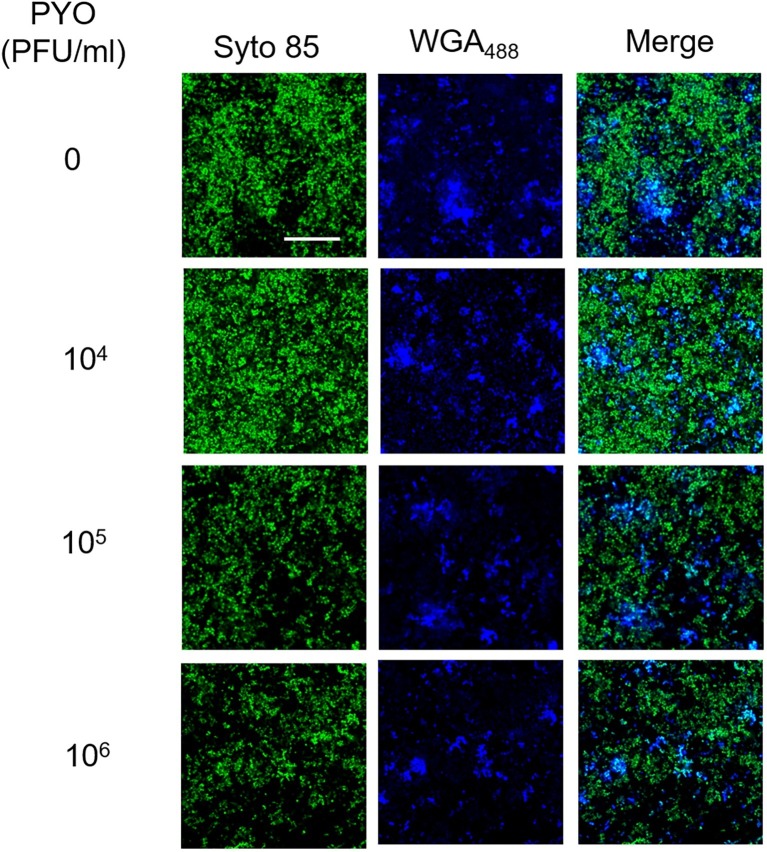
CLSM images of MRSA ATCC 43300 biofilm treated with/without PYO. MRSA biofilm (24 h old) was exposed for 24 h to different PYO titers (ranging from 0 to 10^6^ PFU/ml) and then stained with WGA488 (488/500–600 nm – blue) for exopolysaccharides and SYTO™85 (561/600–700 nm – green) for bacterial cells. Scale bar: 25 μm.

In our previous work, we have shown that Sb is able to degrade the extracellular polysaccharide matrix of *S. aureus* biofilm (Tkhilaishvili et al., 2018b). By using CLSM, we also assessed the effect of PYO on extracellular matrix (Figure 4). Twenty-four-hour-old biofilm of MRSA was stained with both a dye specific for the poly-N-acetylglucosamine residues (blue) of the extracellular polysaccharides and with syto 85 specific for the cellular DNA (green). In contrast to what observed for Sb (Tkhilaishvili et al., 2018b), none of PYO titers determined a progressive reduction of the blue staining in comparison to the untreated control, suggesting that no visible degradation of the polysaccharide component seems to occur in the presence of PYO phages.

**Figure 5:**
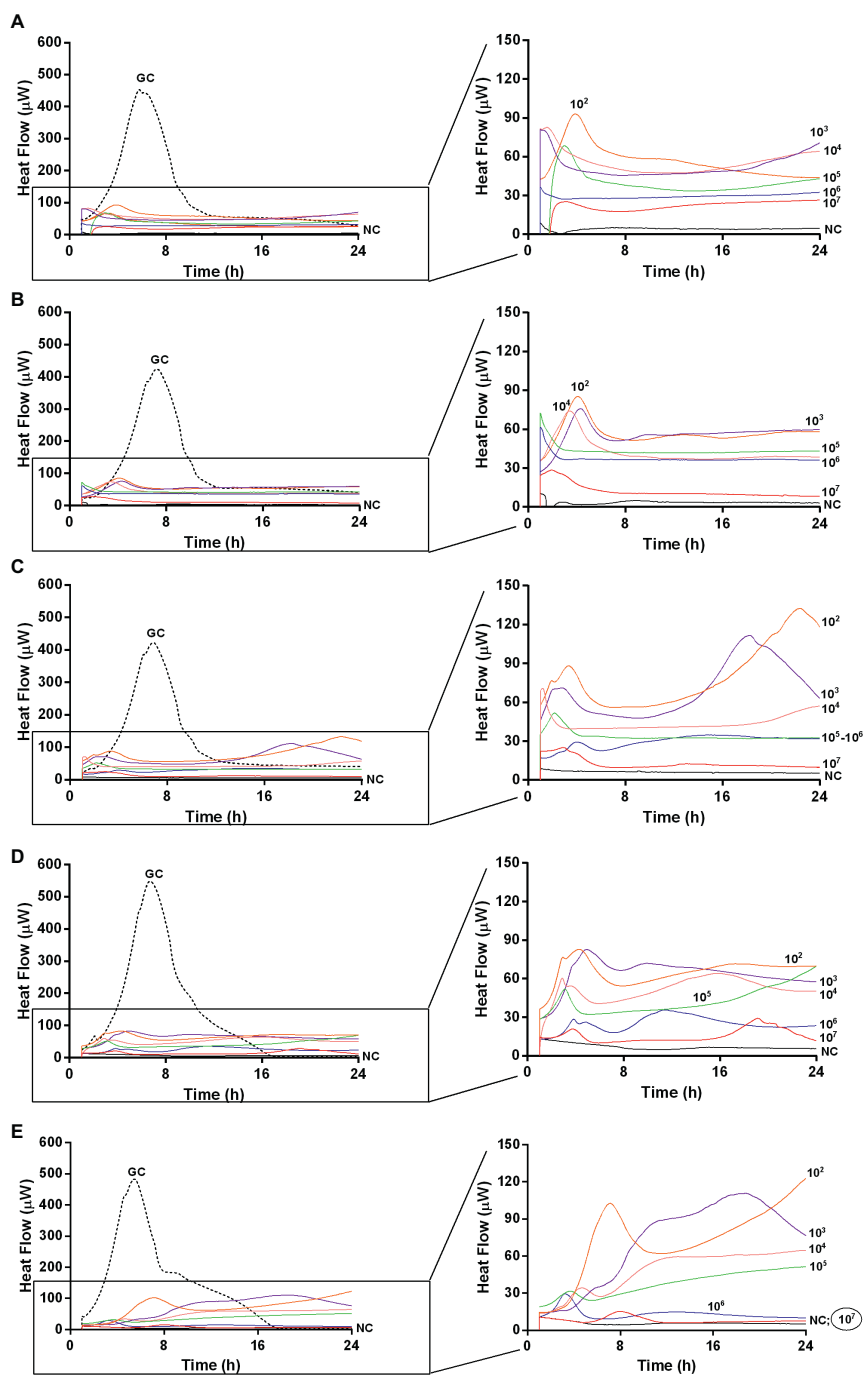
Evaluation of MRSA ATCC43300 biofilm susceptibility to Sb phage at different time exposures by IMC. Each curve shows the heat produced by viable bacteria present in the biofilm after 24 h **(A)**, 48 h **(B)**, 72 h **(C)**, 120 h **(D)**, 168 h **(E)**, treatment with different phage titers (ranging from 10^2^ to 10^7^ PFU/ml). Graphs on the right represent the magnification of the graphs on the left. Numbers above curves represent Sb titers (in PFU/ml). Circled values represent the MBEC. GC, growth control (dashed line); NC, negative control.

**Figure 6:**
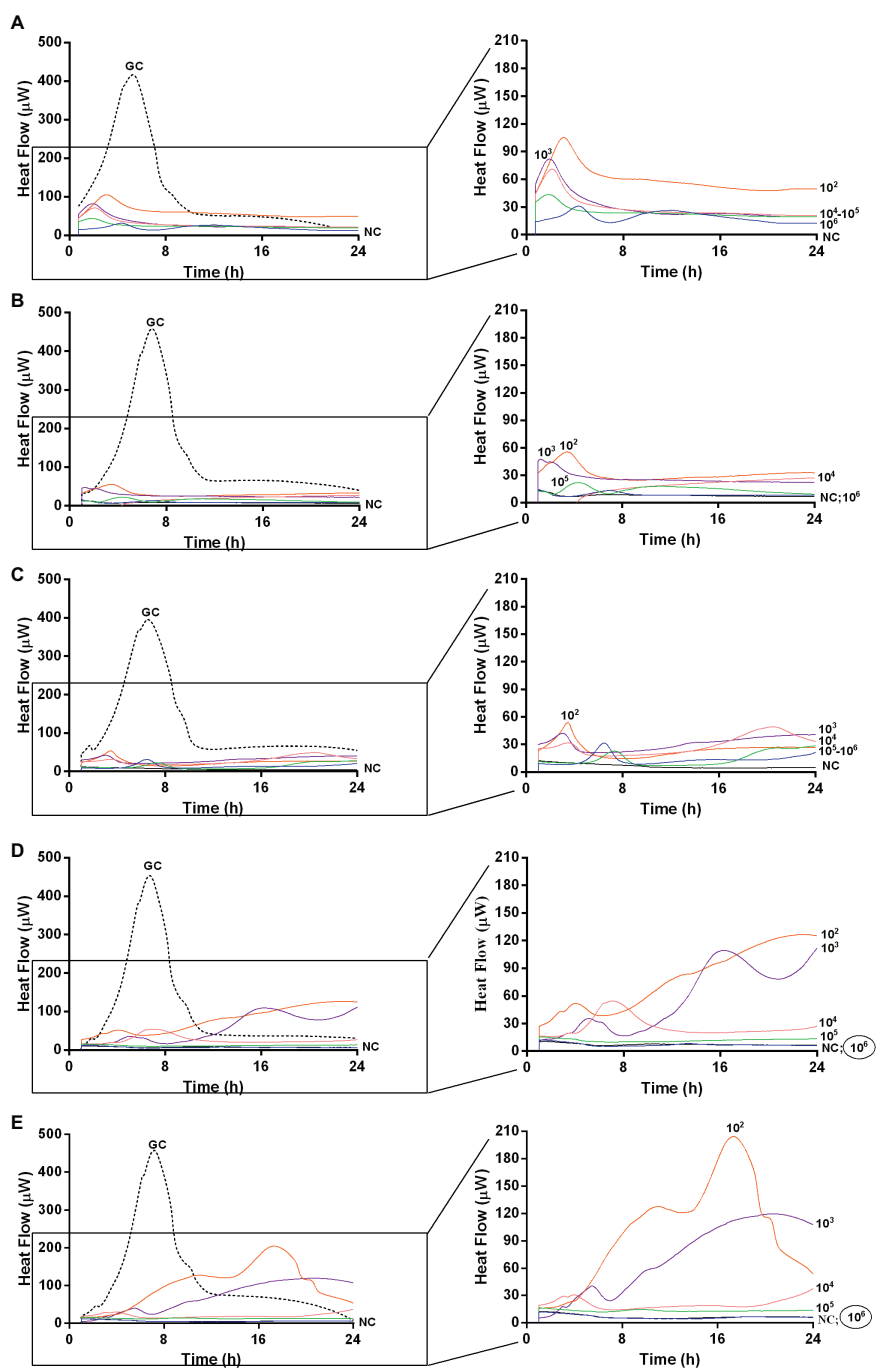
Evaluation of MRSA ATCC43300 biofilm susceptibility to PYO at different time exposures by IMC. Each curve shows the heat produced by viable bacteria present in the biofilm after 24 h **(A)**, 48 h **(B)**, 72 h **(C)**, 120 h **(D)**, 168 h **(E)** treatment with different phage titers (ranging from 10^2^ to 10^7^ PFU/ml). Graphs on the right represent the magnification of the graphs on the left. Numbers above curves represent PYO titers (in PFU/ml). Circled values represent the MBECP. GC, growth control (dashed line); NC, negative control.

Moreover, isothermal microcalorimetry was used to investigate the presence of residual biofilm cells attached on glass beads in terms of metabolic heat production. Beads were re-inoculated in fresh medium, after incubation with different phage titers and at different time points (ranging from 24 h up to 168 h). Indeed, co-incubation with increasing titers of either Sb and PYO formulations over the time led to a minor heat production compared to the heat released by untreated controls, suggesting a strong decrease in the number of alive bacteria attached on the beads when treated with phages (Figures 5, 6, respectively).

**Figure 7:**
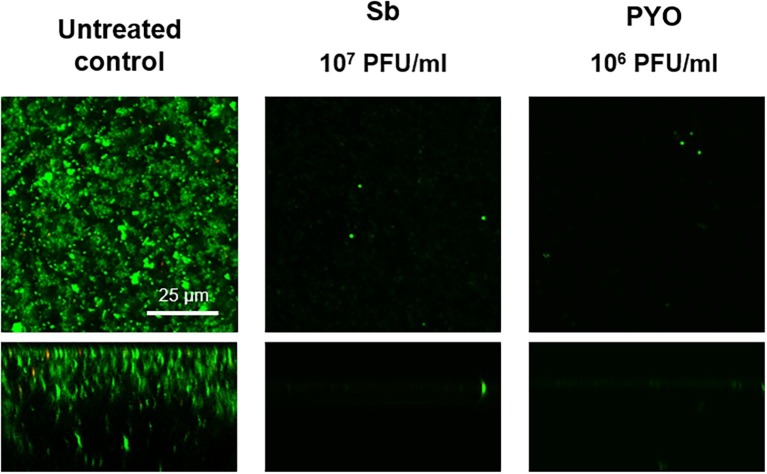
CLSM images of MRSA ATCC 43300 biofilm untreated and treated with Sb and PYO. MRSA biofilm (24 h-old) was exposed for 7 and 5 days to 10^7^ PFU/ml Sb and 10^6^ PFU/ml PYO, respectively. The viability of the cells was evaluated staining with green fluorescent labeled SYTO9 (488/500–540 nm) for alive bacteria and with red fluorescent propidium iodide (PI) (561/600–650 nm) for dead bacteria. Images are merged from the two channels. Upper and lower panels represent xy- and z-plans, respectively. Scale bar: 25 μm.

The analysis of sonication fluids of bead biofilms pre-treated with phages and incubated in the calorimeter at 37°C for 24 h in fresh medium revealed that MRSA biofilm was eradicated only following a co-incubation with the highest titer of Sb and PYO, for either 7 or 5 days, respectively (Figures 5E, 6D). These results were confirmed by CLSM imaging of MRSA biofilm treated for 7 and 5 days with Sb and PYO, respectively. The analysis of MRSA viability by dead/live staining clearly indicating that no biofilms were detectable after the treatment of MRSA biofilm with phages (Figure 7). By definition, 10^7^ PFU/ml and 10^6^ PFU/ml were the MBEC of Sb and PYO after 7 and 5 days, respectively.

### Ability of Sb and PYO to Prevent Methicillin-Resistant *Staphylococcus aureus* Biofilm Formation

**Figure 8:**
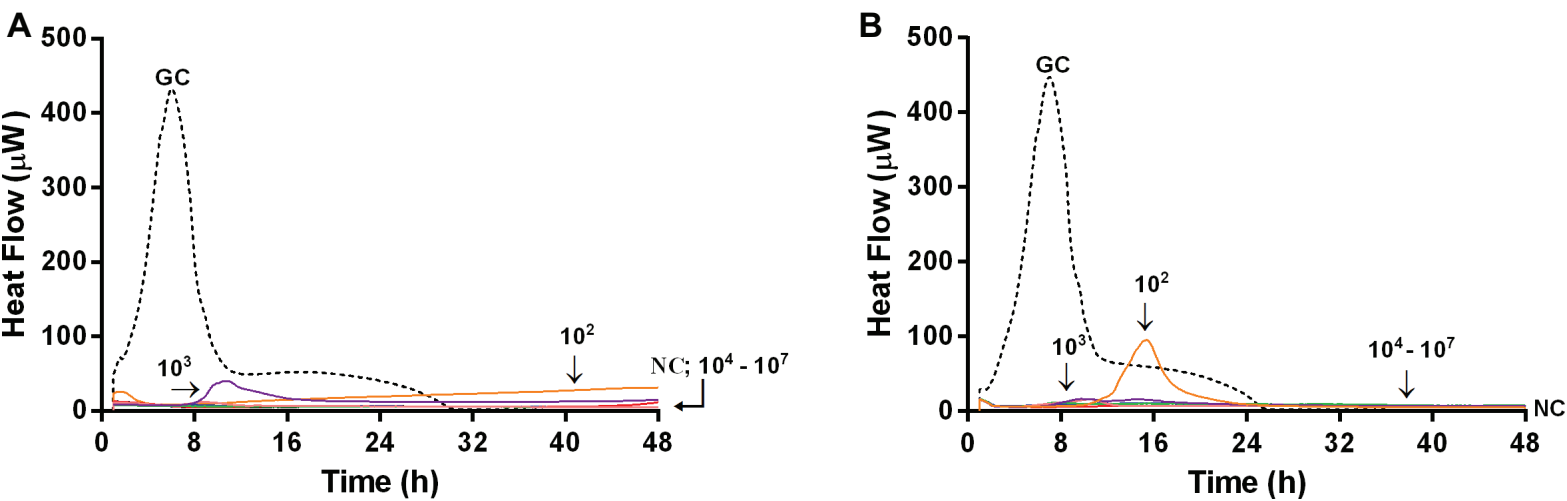
Evaluation of the ability of Sb **(A)** and PYO **(B)** phages to prevent biofilm formation on porous glass beads by IMC. Each curve shows the heat produced by viable bacteria potentially attached on the glass beads after 24 h co-incubation with increasing titers of phages (ranging from 10^2^ to 10^7^ PFU/ml) in the presence of the abiotic surface. Numbers above curves represent Sb titers (in PFU/ml). GC, growth control (dashed line); NC, negative control.

Microcalorimetric measurements were also performed to evaluate the ability of bacteriophages in preventing biofilm formation on glass beads. In this experiment, by using microcalorimetry, we aimed at evaluating if any biofilm or attached bacteria were present on the beads which were previously co-incubated with phages and bacteria (simultaneously), for 24 h. After co-incubation, beads were washed to remove free floating phages and bacteria and then inoculated in fresh medium in the calorimeter. If phages prevented bacterial attachment and biofilm formation, beads had no bacteria on top, so no heat was produced within 48 h of measuring. Figure 8 shows the heat flow detected during 48 h monitoring of heat produced by MRSA, generated by viable bacteria attached on the beads, previously co-incubated with either Sb (Figure 8A) or PYO phage (Figure 8B).

A strong reduction of MRSA heat production was already achieved at a lower titer (10^2^ PFU/ml) of both bacteriophages, and a reduction of more than 90% of heat production was already observed at 10^4^ PFU/ml of both phage formulations. The lack of heat production for 48 h correlated with no biofilm-embedded cells attached on porous surface of glass beads.

**Figure 9:**
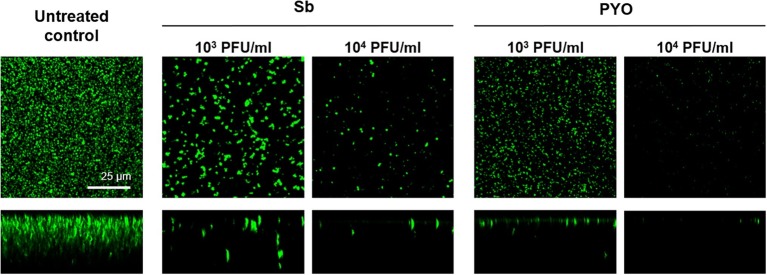
CLSM analysis of biofilm of MRSA ATCC 43300 preventively incubated with either Sb or PYO. The viability of the cells attached on the surface was evaluated after 24 h incubation staining with green fluorescent labeled SYTO9 (488/500–540 nm) for alive bacteria and with red fluorescent propidium iodide (PI) (561/600–650 nm) for dead bacteria. Images are merged from the two channels. Upper and lower panels represent xy- and z-plans, respectively. Scale bar: 25 μm.

These results were confirmed by CLSM analysis of the presence of biofilm MRSA, whose planktonic cells were previously incubated with phages and then labeled by dead/live staining. As shown in Figure 9, a strong reduction or the absence of the alive bacterial cells attached on the wilco surface after co-incubation with 10^3^ PFU/ml and 10^4^ PFU/ml, of both phages compared to the untreated control was observed.

**Figure 10:**
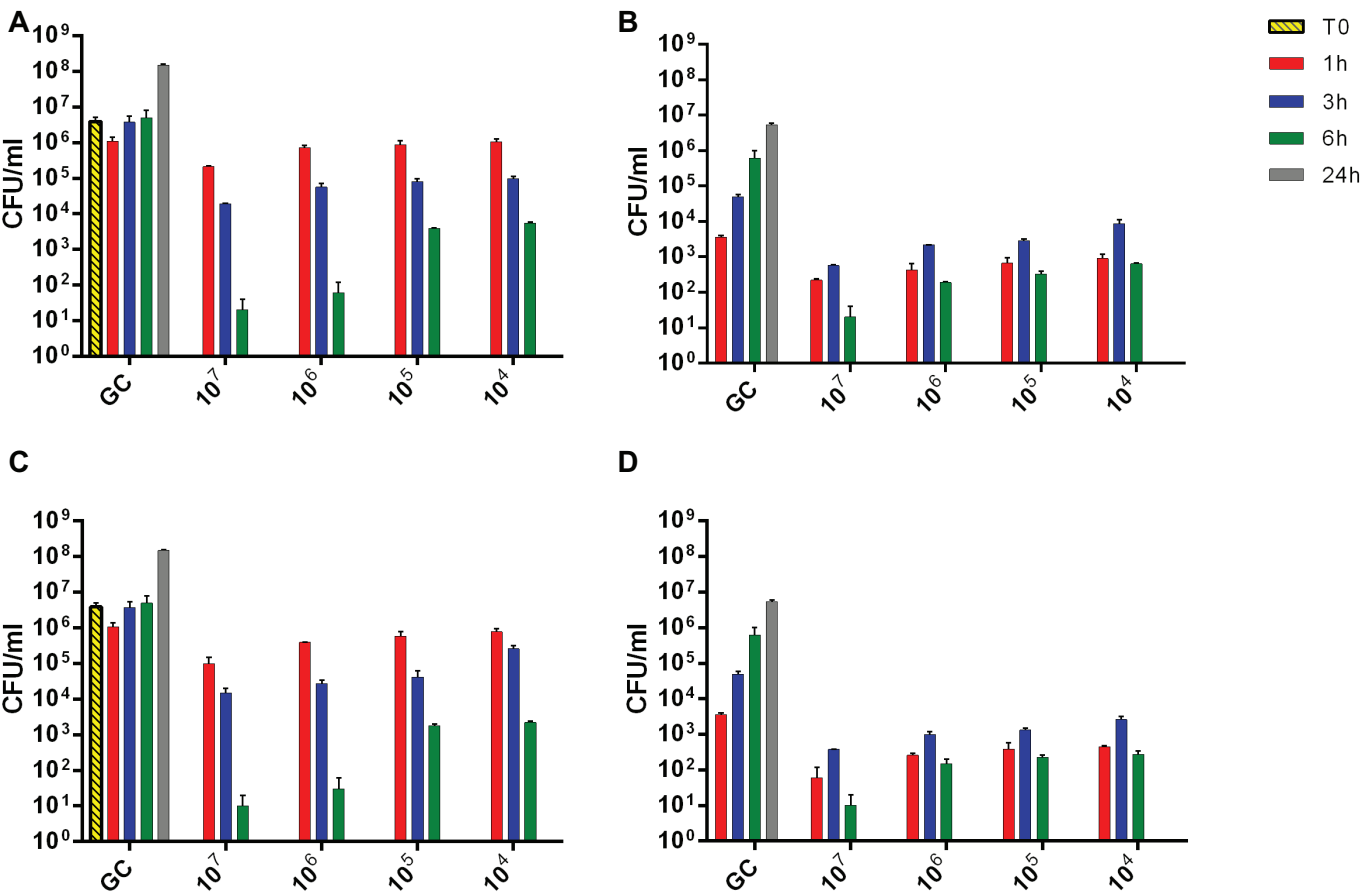
The evaluation of viable bacteria attached on the beads **(B,D)** or free-swimming bacteria **(A,C)** in the supernatant. Histogram represents the mean of CFU number ± SEM of biofilm MRSA treated/untreated with different titer of Sb **(A,B)** and PYO **(C,D)** phages (ranging from 10^4^–10^7^ PFU/ml). GC, growth control.

In order to better understand the absence of heat production detected by microcalorimetric experiments, the evaluation of viable bacteria attached on the beads or free-swimming bacteria in the supernatant was performed by colony counting of either sonication fluids or supernatants. As shown in Figure 10, sub-inhibitory titers (10^4^ PFU/ml) of both phages formulations within 6 h of co-incubation showed a reduction of more than 3 log_10_ of free-floating bacteria in the liquid medium, besides ≈10^2^ CFU/ml of bacteria was attached on the beads. However, after 24 h of co-incubation within phages, no colonies were detected, neither in the liquid medium nor in the sonication fluid. A dose-dependent correlation was observed during colony counting of bacteria with increasing the titer of phages.

### Prophylaxis and Treatment of Methicillin-Resistant *Staphylococcus aureus* Systemic Infection in *Galleria mellonella* Larvae by Either Sb or PYO Phages

**Figure 11:**
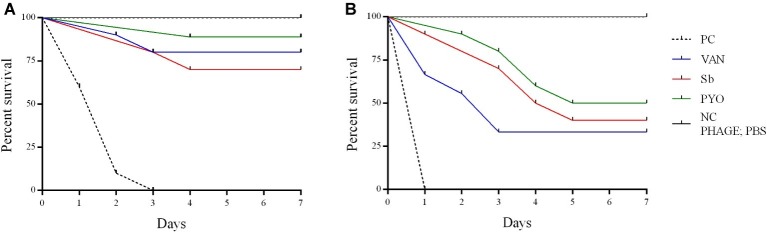
Impact of single phage and vancomycin doses on treatment of MRSA infection and survival rates of *G. mellonella larvae*. A single phage dose (10^5^ PFU/ml) was injected 1 h before the larvae was infected with **(A)** 2.5–5 × 10^6^ CFU/ml **(B)** 2.5–5 × 10^7^ CFU/ml of bacteria. PC, untreated control; NC, non-manipulated; PBS, phosphate-buffered saline.

**Figure 12:**
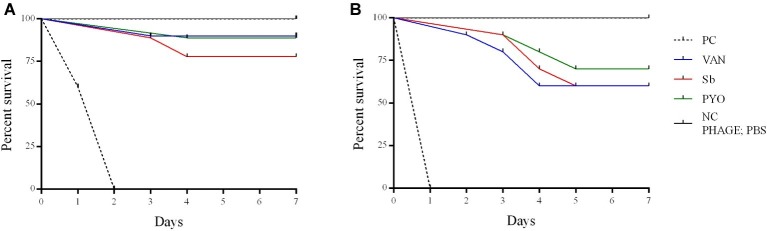
Impact of single phage and vancomycin doses on prevention of MRSA infection and survival rates of *G. mellonella larvae*. A single phage dose (10^5^ PFU/ml) was injected 1 h before the larvae was infected with **(A)** 2.5–5 × 10^6^ CFU/ml **(B)** 2.5–5 × 10^7^ CFU/ml of bacteria. PC, untreated control; NC, non-manipulated; PBS, phosphate-buffered saline.

To evaluate the application of the phages as prophylactic or remedial treatment for MRSA infection, we determined their efficacy in the *G. mellonella* larvae using the same bacterial strain and phages combination used for the *in vitro* assays (Figures 11, 12). Two models of phage therapy were examined. The first was a treatment whereby an acute 1 h infection was allowed to establish prior to administration of phage or vancomycin (Figure 11); in the second model, a phage formulation or vancomycin was administered to larvae 1 h prior to bacterial challenge to prevent infection (Figure 12). No mortality was recorded in the negative controls. Moreover, phages (10^5^ PFU/ml) and vancomycin 10 mg/kg alone had no effect on larval survival, as compared to the PBS-treated controls and were therefore considered to be non-toxic at these doses. Larval survival was affected by the inoculum dose, with higher doses of bacteria (2.5 × 10^7^ CFU/ml) reducing the time of larval survival (Figures 11B, 12B). Single treatment with different doses of phages or antibiotic administered before or after inoculation with MRSA improved larval survival. However, the groups with a lower inoculum (2.5 × 10^6^ CFU/ml) had a greater survival percentage (Figures 11A, 12A). In addition, the results showed that increased survival rate was observed with the usage of a phage cocktail, as compared to single phage administration.

## Discussion

The emergence of antibiotic resistance has reduced the available treatment options for bacterial infections (O’Connell et al., 2013; Li and Webster, 2018). Moreover, the ability of *S. aureus* to form biofilm in the presence of a medical indwelling device represents an additional challenge, since only few antibiotics, such as vancomycin, daptomycin, fosfomycin, and rifampicin, result effective for the treatment of staphylococcal biofilm-associated infections (Singh et al., 2010; Mihailescu et al., 2014). In most cases, biofilm bacteria are tolerant to high concentrations of antibiotics due to the presence of persisters, a subpopulation of bacterial cells phenotypically tolerant to antibiotics, determined the need of prolonged therapies (Lebeaux et al., 2014; Butini et al., 2019). As a result, bacteriophages have been re-emerged as potential alternative strategy for the treatment of biofilm-associated infections (Wu et al., 2015; Pires et al., 2017).

Among different commercially available phage formulation, Sb is constituted of Sb-1 a fully sequenced single phage preparation, while PYO is a cocktail of different phages and the one used in this study contained the completely sequenced Staphylococcal phage ISP (Vandersteegen et al., 2011).

In our previous work, by using IMC, we observed that T3 phage exerted killing activity against planktonic and biofilm-embedded *E. coli* in a titer dependent manner and the highest titer tested (10^7^ PFU/ml) was able to kill all planktonic cells, but it was not able to eradicate sessile bacteria after 24 h incubation (Tkhilaishvili et al., 2018a). A similar trend was observed here. Microcalorimetric analysis in real-time showed that the incubation with 10^7^ PFU/ml titer of either Sb or PYO exhibited a killing activity versus sessile cells of *S. aureus* ATCC43300, but it did not result in an eradication of the biofilm within 24 h, as attested even by colony counting and CLSM imaging. When we compared the biofilm untreated control (growth control) to the biofilm samples treated with the highest titers of phages tested for 24 h, we observed that 90% of heat reduction of the curves in calorimetric graphs corresponds to a moderate reduction of *S. aureus* viability (≈2 log_10_ and ≈3.5 log_10_ CFU/ml) for Sb and PYO treatments, respectively. This is consistent with the fact that the detection limit of the calorimeter is ≈10^5^ CFU/ml (Butini et al., 2019), therefore in order to better quantify the remaining bacterial cells on glass beads the colony counting is needed.

Bacteriophages, as all viruses, infect their host cells to replicate themselves and release the viral progeny, meaning an exponential increase of the number of virus particles over time (Yin and Redovich, 2018). In addition, in our previous work, we showed that phages can also kill persister cells after their resumption to a normal growing phenotype (Tkhilaishvili et al., 2018b). Therefore, we evaluated if a longer exposure could eradicate all sessile bacteria. We observed that the *in vitro* killing of all *S. aureus* biofilm-embedded cells was possible when bacteria were incubated over 5 and 7 days with either PYO (10^6^ PFU/ml) or Sb (10^7^ PFU/ml), respectively. A prolonged exposure characterized by a rise of phage number during bacterial lysis might facilitate the interaction by phages with bacterial cells, including those localized into the deepest biofilm layers, which determine consequently the death of all the adherent cells.

In a previous work, we also showed that Sb can degrade the exopolysaccharide component of the matrix. Here, the CLSM analysis showed that the treatment of biofilm based on PYO had no effect on the extracellular components of the matrix suggesting a different enzymatic activity between the phage formulation tested. However, the slower lytic activity of phages against biofilms can be apparently more due to the physiological state of sessile community and not to diffusion barriers (Abedon, 2017).

As an established biofilm is difficult to be eradicated, the prevention of the infection is still considered the best strategy to reduce the cases of infections (Chen et al., 2013). Either Sb or PYO showed an *in vitro* ability to prevent *S. aureus* biofilm formation within 24 h at titer (10^4^ PFU/ml) lower than that needed for the killing of all planktonic cells of the initial inoculum as shown in Figure 1. In such experiment, we observed only a 2 log_10_ CFU reduction of bacteria treated with 10^4^ PFU/ml comparing to the untreated control. This discrepancy might be due to the fact that in the presence of the beads part of the planktonic bacteria attaches on the material, resulting less metabolically active. This effect reduces the number of the replicating free-floating bacteria and be more easily lysate by phages. This effect was observed for both phages formulation tested independently. As reported by *in vitro* observations, the adhesion of *S. aureus* seems to occur within 3 h from bacterial inoculum (Moormeier et al., 2014). CFUs counting after 3 h-incubation of *S. aureus* treated with 10^4^ PFU/ml of either Sb or PYO showed that there is no difference in the CFU number of attached cells from treated and untreated samples, suggesting that Sb and PYO did not interfere with bacterial adhesion process. Although we cannot clearly discriminate between biofilm prevention and biofilm disruption, here mature biofilm disruption could be excluded by the fact that the phage titer 10^4^ PFU/ml (determining bacteria eradication on the beads) had only a poor effect in disrupting the biofilms in the “antibiofilm activity test” (Figure 2).

*In vivo* experiments, performed in a *G. mellonella* model of *S. aureus* systemic infection, showed that both phage formulations at higher titer tested improved the survival of larvae comparing to the untreated sample and analogously to the effect exhibited by vancomycin used as antibiotic control.

Previous *in vivo* studies of the therapeutic potential of phages against different bacterial pathogens have used in a *G. mellonella* infection model (Desbois and Coote, 2011; Johnston et al., 2016; Silva et al., 2017). However, to the best of our knowledge, this is the first study to test phages for their ability to rescue MRSA-infected *G. mellonella* larvae from death. In our study, the phage was applied at 1 h before or after MRSA infection in order to determine if these phage formulations had a prophylactic and/or treatment effect. Results suggest that the time of administration of phage plays an important role in therapy of MRSA infection in larvae. When phages were applied 1 h before infection, the survival rates were higher than in the groups administered phage after 1 h infection. In addition, our study showed that the effectiveness of phage therapy increased with usage of phage cocktail compared to single phage and a single phage dose was enough to reduce the mortality of larvae infected with MRSA *in vivo*. The low MOI (0.1 and 0.01) used in this study allowed for determination of the efficacy of the phages while avoiding the phenomenon of “lysis from without.” Interestingly, a MOI 0.1 determined the survival of more larvae comparing to the larval survival with a MOI 0.01, suggesting that the ratio between phages and bacteria is relevant for the success of the therapy. However, the relevance of the *G. mellonella* model to predict the phage efficacy with higher MOIs and different timings of phage administration remains to be determined.

## Conclusion

In this study, our research strongly suggests that phage PYO bacteriophage and Sb phages are promising for preventing device colonization and killing biofilm bacteria attached on a surface. Novel strategies for addressing coating and release of phages from material should be further investigated. In addition, both phage formulations increased the survival of *G. mellonella* larvae preventing or treating MRSA infection compared to untreated control. Future work should be considered using the phages against clinical strains and efficiency of treatment for systemic infections must be evaluated in more complex animal models.

## Data Availability Statement

The datasets generated for this study are available on request to the corresponding author.

## Author Contributions

TT, MD, and ATr conceived and designed the experiments. TT and MD performed the experiments, analyzed the data, and drafted the manuscript, with the contribution of ATr, LW, and ATa.

### Conflict of Interest

The authors declare that the research was conducted in the absence of any commercial or financial relationships that could be construed as a potential conflict of interest.
